# New Insights into Molecular Mechanisms of Immune Complex-Induced Injury in Lung

**DOI:** 10.3389/fimmu.2016.00086

**Published:** 2016-03-09

**Authors:** Peter A. Ward, Fatemeh Fattahi, Markus Bosmann

**Affiliations:** ^1^Department of Pathology, University of Michigan Medical School, Ann Arbor, MI, USA; ^2^Center for Thrombosis and Hemostasis, University Medical Center, Mainz, Germany

**Keywords:** extracellular histones, neutrophil extracellular traps, complement, C5a, C5aR1, C5aR2, acute respiratory distress syndrome

## Abstract

While the phlogistic activities of IgM or IgG immune complexes (ICs) have been well established as complement-activating agents and seem likely to play important roles in humans with vasculitis, certain types of glomerulonephritis as well as in a variety of autoimmune diseases, the predominant clinical strategies have involved the use of immunosuppressive or anti-inflammatory drugs. Over the past decade, new insights into molecular events developing during IC models in rodents have identified new phlogistic products that may be candidates for therapeutic blockade. Extracellular histones, located in the web-like structures of neutrophil extracellular traps, are released from complement-activated polymorphonuclear neutrophils (PMNs) downstream of IC deposition. Extracellular histones appear to be a new class of highly tissue-damaging products derived from complement-activated PMNs. Histones have also been discovered in cell-free broncho-alveolar lavage fluids from humans with acute respiratory distress syndrome (ARDS). Recent studies emphasize that in the setting of ARDS-like reactions in rodents, extracellular histones are released and are exceedingly proinflammatory, tissue damaging, and prothrombotic. Such studies suggest that in humans with ARDS, extracellular histones may represent therapeutic targets for blockade.

## Introduction

It has been known for at least 50 years that formation of immune complexes (ICs) (involving IgM or IgG) results in vascular and renal damage that is complement dependent (1–3). In humans, the most common conditions associated with ICs are autoimmune diseases, such as rheumatoid arthritis and systemic lupus erythematosus (SLE), and certain types of glomerulonephritis (4–9). Viral hepatitis C and HIV infections can result in mixed cryoglobulinemia and vasculitis secondary to IC formation (10, 11). In addition, lung diseases, such as acute respiratory distress syndrome (ARDS) and acute lung injury (ALI), are reported to be associated with IC deposition (3, 12). ICs are defined as antigen–antibody complexes formed by ligation of IgG or IgM to soluble antigen, triggering complement activation mainly *via* the classical pathway. ICs bind to activating and/or inhibitory Fcγ receptors (FcRs) to facilitate phagocytosis and to modulate immune cell functions (13–15). ICs activate the complement system as an integral arm of innate immunity, which results in the formation of the C5b-9 membrane attack complex and generation of anaphylatoxins (C3a and C5a) (16). Over the past several years, important new information has been obtained that expands our knowledge of the molecular events that are linked to IC-induced injury. This review will focus on the functional ties of IC with the complement system and extracellular histones, which together with DNA and products of neutrophil granules are major components of neutrophil extracellular traps (NETs).

## Model of Acute Lung Injury Induced by IgG Immune Complexes

**Figure 1:**
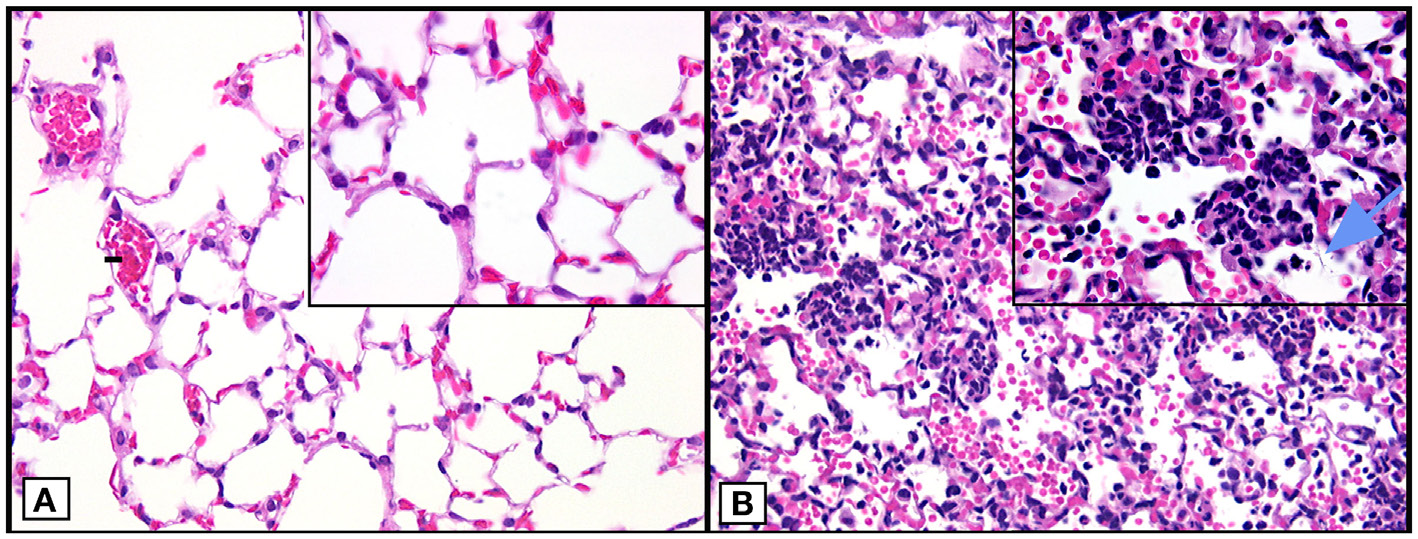
**Histopathology of mouse lungs in normal lungs and lungs after ALI following airway deposition of IgGIC in C57BL/6J mice**. **(A)** Histological features of normal mouse lung. **(B)** Positive control (acutely injured lung induced by airway deposition of IgGIC) 6 h after induction of ALI showing lung infiltration of PMNs, intra-alveolar hemorrhage, and hyaline membranes (arrow). Images modified from Bosmann et al. (18). Both frames are from paraffin-embedded sections stained with hematoxylin and eosin (×40 view; insets: ×100 view).

Our focus over the past several years has been related to the finding of extracellular histones in the setting of IgGIC-induced ALI in mice and the mechanisms related to how the appearance of extracellular histones play into the events that result in severe compromise of air exchange in the lung (17). The IgGIC–ALI model features the intratracheal (i.t.) instillation of rabbit IgG, which has high reactivity with bovine serum albumin (BSA). BSA is then given intravenously, resulting in IgGIC formation in walls of alveoli and interstitial capillaries of lung. This results in a break in the vascular–endothelial barrier and the alveolar–epithelial barrier, leading to alveolar edema, hemorrhage, and the massive influx of polymorphonuclear neutrophils (PMNs) as shown in Figure 1 (18, 19). In addition, residential CD11c^+^F4/80^+^ alveolar macrophages are actively involved during IgGIC–ALI, which is similar as in other ALI models (20, 21). Together, activated PMNs and macrophages release large amounts of oxidants and proteases, resulting in intense damage to the alveolar wall.

## Immune Complexes Mediate Complement Activation and Appearance of Extracellular Histones

Full expression of the features of injury in the IgGIC model of ALI requires participation of the complement system (3). The complement system is a conserved arm of innate immune defenses. It comprises more than 30 proteins with cascadic proteolytic activation in response to antigen–antibody aggregates (classical pathway), foreign surfaces devoid of endogenous complement inhibitors (alternative pathway), or innate recognition of microbial pathogen surfaces (lectin/ficolin pathway). These three activation pathways converge on the level of C3/C5 to catalyze C5b-9 membrane attack complex formation as a major mechanism of IC-mediated tissue injury. In addition, IgG and IgM antibody–antigen ICs triggered complement activation results in generation of C3a and C5a during ALI. A great deal of research work has focused on C5a because it is considered to be of higher biological activity as compared to C3a (22). Some time ago, we described that i.t. instillation of neutralizing antibodies to rat or mouse C5a had protective effects in the setting of IgGIC-induced ALI (23–25). The surge of C5a in lung homogenates and broncho-alveolar lavage fluids (BALFs) was observed about 6 h after IC formation (26). Anti-C5a neutralizing antibodies were also protective in sepsis-induced ARDS of non-human primates (27). C5a ligates with its two receptors, C5aR1 and C5aR2. In contrast to initial beliefs, the C5aR2 receptor appears to be critically involved in the proinflammatory effects of C5a in lung inflammation. In recent studies, use of mice lacking either C5a receptor (C5aR1 or C5aR2) markedly reduced development of inflammation (19), lung dysfunction and albumin leak into alveoli, buildup of PMNs, as well as the appearance in BALF of proinflammatory cytokines and chemokines (17).

We earlier described the development of intense inflammation and injury in the IgGIC model of ALI in C3-deficient mice (28). This had been reported somewhat earlier by another group in non-lung IC disease models and the initial conclusion was that complement was, therefore, not involved in IC-triggered inflammatory events (29). We reproduced and expanded the earlier data indicating that full inflammatory damage occurred in C3^−/−^ mice after alveolar deposition of IgGIC in mouse lungs. However, we also found that our neutralizing antibody to mouse C5a was highly protective, suggesting a non-traditional source for generation of C5a. We were able to show that specific inhibitors of thrombin (such as antithrombin or hirudin) prevented the development of IgGIC-induced ALI in C3^−/−^ mice (28). C3a is generated during IC-induced inflammation (30), but C3a is less active than C5a in lungs (22, 26). Indeed, C3a may act as an anti-inflammatory modulator in direct opposition to C5a (31). In future, studies with C3aR-deficient mice in IC-induced ALI could be important to obtain a more complete picture. Likewise, the distinctive functional roles of C3b and its proteolytically inactive fragment, iC3b, remain to be determined in IC-induced ALI. Direct *in vitro* experiments with human thrombin and human C5 demonstrated that thrombin was able to generate authentic C5a when incubated with C5. The sequence of the peptide that was generated from C5 by thrombin was identical to that of C5a generated by the complement-derived C5 convertases. This established that C5 convertases unrelated to complement-generated C5 convertases could generate C5a (28). More recent work has demonstrated that some activated clotting factors (such as FIXa, FXa, FXIa, and plasmin) also have the abilities to generate C3a and C5a when incubated with purified human C3 or C5 (32–34). In addition, the T-cell expressed protease cathepsin L may provide a mechanism of intracellular complement activation by cleavage of endosomal and lysosomal C3 into biologically active C3a and C3b (35).

Modern imaging technology including magnetic resonance imaging (MRI), together with transmission electron microscopy (TEM), has been employed in the model of ALI revealing lung consolidation, which correlated to the histological features described above (17, 26). Whole-body plethysmography has allowed measurements of various parameters of respiratory function.

Immune complexes *via* C5a/C5aR1/C5aR2 promote the appearance of extracellular histones, the latter being major components of NETs. Extracellular histones, which were found in BALF, were quantified by Western blotting and ELISA (17). The most convincing evidence that extracellular histones were linked to ALI-induced lung dysfunction came from experiments in which neutralizing antibodies to histones (H2A and H4), which were given i.t. together with recombinant mouse C5a. This experimental design led to significant reduction in the intensity of ALI in mice (17). The lack of additional neutralizing monoclonal antibodies to other histones has prevented more detailed information about the roles of individual histones in development of ALI. Collectively, these data suggest a pathophysiologic sequence of events in which IC deposition promotes complement activation with release of C5a. Subsequently, C5a *via* its C5aR1 and C5aR2 receptors mediates generation of extracellular histones (as a component of NETs) to induce tissue damage. Thereby, C5a may link IC deposition with extracellular histones and NETs during inflammatory diseases and ALI/ARDS in particular.

## Lung Damaging Effects of Extracellular Histones

Based on the *in vivo* data incriminating the roles of extracellular histones in development of ALI following alveolar deposition of IC (see above), we undertook a series of experiments in which purified or recombinant histones were instilled intratracheally in order to determine their ability to induce ALI as defined by morphology, degree of leak of mouse albumin into BALF, as well as presence of PMNs and cytokines/chemokines in BALF. For most of the studies, a histone mix (obtained from calf thymus) consisting of all five histones was first employed, followed by use of purified histones. It became immediately apparent that extracellular histones in the mixture given intratracheally were extremely lung damaging. Morphologically, there was extensive alveolar edema, sloughing of alveolar and bronchiolar epithelial cells, and alveolar hemorrhage. It was also clear that small venules in the lung parenchyma contained numerous thrombi (17).

The airway (i.t.) administration of the histone mix also caused significant disturbances in alveolar–capillary gas exchange (17). For instance, 15 min after airway delivery of histones, arterial pH fell from 7.4 to almost 7.1, indicating severe acidosis. Arterial pCO_2_ rose from 40 to 75 mmHg. Arterial pO_2_ fell from 88 to 50 mmHg, representing severe hypoxia. Arterial saturation fell from 92 to 48%, all indicating severely compromised gas exchange in alveoli (17). Over a period of 6 h, most of these parameters returned to the normal or near normal range, suggesting the reversibility of these dramatic dysfunctions, the explanation of which is not obvious since, on the basis of albumin leak into BALF, damage continued until 8 h, after which albumin leak began to decrease (17). Extracellular histones caused dramatic ventilation changes as indicated by pathological box flow patterns of whole-body plethysmography within minutes after purified histone i.t. administration (17). Respiratory rates rose from 120 to 175/min 6 h after histone exposure. Minute ventilation rose from 160 to 240 ml/min within the same time period. Similar patterns were found for inspiratory flow rates, inspiratory time, expiratory time, and total respiratory cycles (17). In contrast to the parameters for alveolar–capillary gas exchange which returned to normal or near normal 6 h after histone exposure, the whole-body plethysmography values remained defective for 24 h.

## Balf Responses to Intratracheal Administration of Purified Histones

Not surprisingly, airway administration of calf thymus purified, mixed histones resulted in the release of numerous cytokines and chemokines in BALF, which generally peaked after 8 h (17). Proinflammatory peptides in 8h BALF included IL-1β, IL-6, Eotaxin, KC, MCP-1, MIP-1α, MIP-1β, RANTES, and TNFα. Clearly, a large number of proinflammatory peptides appeared after airway instillation of the histone mix, although we do not have information on the activities of individual histones. For most cytokines and chemokines, levels in BALF were between 100 and 1000 pg/ml. The presence of diverse proinflammatory chemokines and cytokines in the lung is likely related to damaging inflammatory responses triggered by these peptides in various amplification loops. What is not known currently are the cellular sources of these peptides. These histone-induced chemokines most likely promote the influx of PMNs and other white blood cells to the alveolar spaces during ALI/ARDS. On the other hand, airway instillation of purified histone extracts in C5aR1-deficient mice as compared to wild type mice produced similar severities of lung injury as evaluated by influx of PMNs and disruption of the alveolar/capillary barrier (17). This observation may suggest that NETs/extracellular histones do not rely on the liberation of C5a (with subsequent C5aR1 signaling) for mediating tissue damage and inflammation.

Taken together, extracellular histones as downstream products of ICs mediate the adverse effects of ALI. It is known that extracellular histones are endogenous ligands for TLR2 and TLR4 receptors (36, 37), which may suggest that these pattern recognition receptors are involved in mediator production downstream of IC-induced inflammation.

## Effects of Purified Histones on Alveolar Epithelial Cell Lines

For these studies, two mouse cell lines, LA-4 and MLE-12, were used (17). They are morphologically and phenotypically similar to alveolar epithelial cells and contain surfactant. Cells were exposed to the histone mix or purified or recombinant H1, H3, and H4 (50 μg/ml) for varying periods of time. Histones induced significant increases in [Ca^2+^]i within the first 30 min of cell exposure to histones. In the case of MLE-12 cells, cell viability was reduced by 60 min of incubation with histones (17). Cytotoxicity was confirmed by release of lactate dehydrogenase (LDH) in cell culture supernatants, especially in the case of H1, H4, and the histone mix. Abundant release of LDH was found within the first 60 min. These data suggest that some histones can cause cytotoxicity of alveolar epithelial cell lines. The extent to which other lung inflammatory cell types, such as PMNs and macrophages, exhibit similar responses to histones remains to be determined. On the other hand, it is well documented that extracellular histones are cytotoxic for endothelial cells during *in vitro* culture (38–40). It is not determined in IgGIC-ALI to what extent there is apoptosis of both epithelial and endothelial cells in lung, but it appears likely.

## Presence of Extracellular Histones in Balf from Humans with Acute Respiratory Distress Syndrome

On the basis of animal (rats and mice) studies indicating that development of ALI features the appearance of extracellular histones in BALF (17), we have also analyzed BALF from humans with ARDS which has many features of animal ALI. ARDS in humans is usually characterized by bilateral radiological lung densities that are associated with severely compromised air exchange in the absence of cardiac etiologies (41, 42). Morphologically, there is alveolar hemorrhage, edema, and large alveolar accumulations of PMNs. In addition, within alveoli is extensive deposition of “hyaline membranes” along alveolar surfaces. The deposits represent dense buildup of fibrin, which contributes to faulty gas exchange. BALFs contain proinflammatory cytokines and chemokines, along with the complement activation product, C5a. The major risk factors for ARDS are pneumonia, sepsis, acute pancreatitis, transfusion reactions, trauma, and many more (42–44). Beyond supportive care, there are no FDA-approved drugs for the treatment of ARDS. Using cryopreserved BALF samples from individuals with normal lung function or individuals with ARDS, extracellular histone content was measured both by ELISA and by Western blot technology (17). Sequential BALF samples were obtained from a few of the ARDS patients. One patient showed extracellular histone H4 presence on days 5, 14, and 21, while the second patient had strong H4 presence only on day 4 but no detectable presence on days 8 and 21. Both H3 and H4 were detected in many of the BALF samples. In general, histone presence in random BALF specimens from ARDS patients was ≥50% on days 0–10, falling to around 30% on days 11–16. It is quite clear that histone presence may persist for as long as 2 weeks, although these data are preliminary and we neither know the full composition of histones in BALF nor their degradation kinetics or how histone presence relates to the clinical status of patients with ARDS.

## Differences Between Animal Studies and Human Immune Complex Diseases

Many studies of IC-mediated pathology have relied on animal models, e.g., with transgenic mice. These models have been invaluable helpful to understand the development and progression of IC-dependent inflammation and lung injury. On the other hand, animal studies have clear limitations when extrapolations are made regarding human diseases. The IgGIC–ALI model of rodents is usually non-lethal, and this is in contrast to 20–40% mortality rates that have been observed in the past for humans (42).

It is important to appreciate that mouse and human FcRs orthologs do not possess similar effector functions and this is further complicated by a misleading nomenclature (13). For example, the functional ortholog of mFcγRIV is hFcγRIIIA and the functional ortholog of mFcγRIII is hFcγRIIA (45). Overall, the species differences in the repertoire of FcRs may also alter the natural course of IC disease. More recently, the development of humanized mouse models for hFcRs has provided a clearer picture. Smith et al. (46) have reported a mouse model in which all murine FcγRs were deleted and human FcγRs were inserted to the mouse genome. When this humanized FcγRs mouse strain was challenged with heat-aggregated human IgG as a model of IC-mediated disease, such transgenic mice displayed a severe anaphylactic decline in body temperature, which was not observed in wild-type control mice (46).

Equipotent cross-reactivity between mouse and human C5a and its receptors does exist (47). In addition, transgenic knock-in mice with a replacement of mouse C5aR1 with the human C5aR1 have been described (48) but have not yet been studied in IgGIC-ALI.

IgGIC-ALI in rodents may mimic some features of antibody-dependent transfusion-related acute lung injury (TRALI) in humans (49). TRALI develops following transfusion of blood products containing antibodies against MHC class I and human neutrophil antigen (HNA). This promotes acute antibody-dependent recognition of PMNs and endothelial cells in lungs. In addition, formation of IC of anti-MHC class I and anti-HNA antibodies with their soluble antigens may occur and trigger PMN activation *via* FcRs (50). Overall, the contribution of IC in human lungs during the natural development of TRALI may not be as important as during experimental IgGIC-ALI in rodents. On the other hand, the relevance of IC in human diseases, such as the cryoglobulinemia syndrome, serum sickness-like reactions, vasculitis, membranoproliferative glomerulonephritis, and rapidly progressive glomerulonephritis, is well established (2).

## Summary of Molecular Events in Immune Complex-Induced Acute Lung Injury

**Figure 2:**
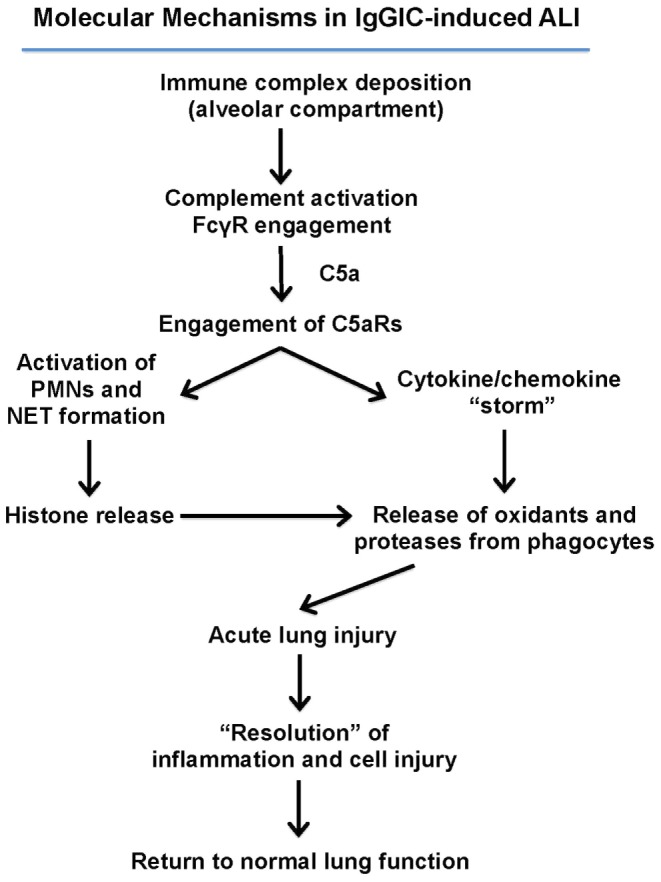
**Molecular mechanisms in IgGIC-induced ALI**. Airway position of IgG immune complexes leads to complement activation and generation of C5a. C5a interacts with its receptors C5aR1 and C5aR2 on neutrophils and on other cell types in lung (alveolar epithelial cells, endothelial cells, and macrophages) resulting in a surge of proinflammatory cytokines and chemokines. These interactions with neutrophils results in the formation of neutrophil extracellular traps (NETs), resulting in the release of extracellular histones, which are extremely cell-toxic, proinflammatory, and thrombogenic. These responses resolve and there is little evidence of residual functional or structural damage.

Figure 2 summarizes our current concepts of molecular events responsible for ALI triggered by alveolar deposition of IgGIC. Similar events were found with mice in which ALI was induced by bacterial lipopolysaccharide (LPS) or recombinant C5a (26).

The IC interact with activating (type I family) FcγRs which are present on PMNs, macrophages, B cells, and other immune cells (51). FcγRs mediate phagocytosis of IC with subsequent antigen processing and major histocompatibility complex-dependent presentation of antigens by professional antigen presenting cells (51, 52). Activated FcγRs will result in the amplification of the early proinflammatory events driving intense lung injury. The effects of IC on immune responses largely depend on the type of engaged FcγRs (e.g., the activating FcγRI, FcγRIIA, FcγRIIC, FcγRIIIA, FcγRIIIB receptors, and the inhibitory receptor FcγRIIB) (52). The recognition of IC by individual FcγRs is mainly determined by IgG subclasses and Fc-antibody glycosylation (52).

Early events in IgGIC-ALI also trigger complement activation, presumably *via* the classical pathway, resulting in generation of C5a. C5a interacts with its receptors, C5aR1 and C5aR2, which exist in large numbers in or on PMNs (53). Much lower numbers of binding sites also exist on endothelial cells and macrophages. In the case of PMNs activated by C5a, this leads to formation of NETs (54). NETs contain extracellular strands of DNA and various proinflammatory proteins (myeloperoxidase, proteases, and histones) that have substantial injurious effects on a variety of cells in the lung (55). In the meantime, large numbers of proinflammatory peptides appear in BALF (described above). These mediators cause lung phagocytes to release oxidants and proteases that damage a variety of resident cells in lung. Rather surprisingly, the IgGIC model in rodents progresses to “resolution” (disappearance of inflammatory cells and mediators), resulting in return of the lung to normal morphology and function. This is in contrast to ARDS in humans, where mortality rates are at least around 20% despite critical care, supportive treatments. ARDS in humans may lead to fibrosis alveolitis (56). Very little is known about how the lung can sustain severe inflammatory injury without experiencing evidence of permanent structural and functional derangements. It is possible that mesenchymal or hematopoietic stem cells might be responsible for recovery (57).

## Conclusion

It is clear that following alveolar deposition of IgGIC, C5a, or LPS, ALI develops in a manner that is C5a-dependent and C5a receptor-dependent. In these models, PMN activation occurs, resulting in NET formation and resultant release of extracellular histones together with PMN products, such as proteases, myeloperoxidase, and oxidants, that are lung damaging. The preliminary data in patients with ARDS confirms the presence of extracellular histones in BALF. The experimental evidence suggests that extracellular histones are highly proinflammatory and thrombogenic in the lung. A constraint in full understanding of such events is the current lack of quantitative ELISA technology that will reliably measure amounts of individual histones and NET-predisposing histone modifications. Versions of mass spectometry can identify the presence of individual histones using laborious technology, but in such situations, mass spectrometry is largely non-quantitative. It is imperative that reliable ELISA technology be developed.

In addition, we still know very little about the molecular pathways of post translational modifications of histones, such as conversion of arginine residues in histones to citrullination products. It is known that such changes, caused by peptidyl arginine deiminase 4 (PAD4), converts arginine residues to citrullinated residues which may confer autoimmune epitopes on histones, resulting in autoantibodies which may exacerbate autoimmune diseases, such as small-vessel vasculitis and SLE (58, 59). Very little is known about how such post-transitional modifications of histones may affect biological activities of histones. In addition, the precise intracellular pathways by which IC trigger NET formation and histone release remain to be determined, but most likely involve FcγRs. IC may contain nucleic acids from dead cells (such nucleic acids are also a major component of NETs) for modulation of type I interferon-dependent immune responses (60). Accordingly, much work needs to be done to better understand the pathobiology of histones and to connect all the dots that link extracellular histones and NETs with IC-mediated tissue damage.

## Author Contributions

The manuscript was written by PW and MB with the assistance of FF.

## Conflict of Interest Statement

The authors declare that the research was conducted in the absence of any commercial or financial relationships that could be construed as a potential conflict of interest.
